# Innate Immunity Holding the Flanks until Reinforced by Adaptive Immunity against *Mycobacterium tuberculosis* Infection

**DOI:** 10.3389/fmicb.2016.00328

**Published:** 2016-03-14

**Authors:** Nargis Khan, Aurobind Vidyarthi, Shifa Javed, Javed N. Agrewala

**Affiliations:** ^1^Council of Scientific and Industrial Research – Institute of Microbial TechnologyChandigarh, India; ^2^Department of Cytology and Gynecologic Pathology, Postgraduate Institute of Medical Education and ResearchChandigarh, India

**Keywords:** *Mycobacterium tuberculosis*, innate immunity, apoptosis, autophagy, nitric oxide, inflammasome

## Abstract

T cells play a cardinal role in imparting protection against *Mycobacterium tuberculosis (Mtb)*. However, ample time is required before T-cells are able to evoke efficient effector responses in the lung, where the mycobacterium inflicts disease. This delay in T cells priming, which is termed as lag phase, provides sufficient time for *Mtb* to replicate and establish itself within the host. In contrast, innate immunity efficiently curb the growth of *Mtb* during initial phase of infection through several mechanisms. Pathogen recognition by innate cells rapidly triggers a cascade of events, such as apoptosis, autophagy, inflammasome formation and nitric oxide production to kill intracellular pathogens. Furthermore, bactericidal mechanisms such as autophagy and apoptosis, augment the antigen processing and presentation, thereby contributing substantially to the induction of adaptive immunity. This manuscript highlights the role of innate immune mechanisms in restricting the survival of *Mtb* during lag phase. Finally, this article provides new insight for designing immuno-therapies by targeting innate immune mechanisms to achieve optimum immune response to cure TB.

## Introduction

Tuberculosis continues to affect public health worldwide. About one third of the global population is infected with *Mtb* but only 3–10% succumb to disease (Barry et al., 2009; Ottenhoff and Kaufmann, 2012). Therefore, greater than 90% of infected population remains asymptomatic, which determines the intricate balance between host immunity and *Mtb*. Understanding the immune response of these asymptomatic individuals can be highly informative and will provide potentially new pathways for the development of anti-TB drugs and vaccines.

**FIGURE 1:**
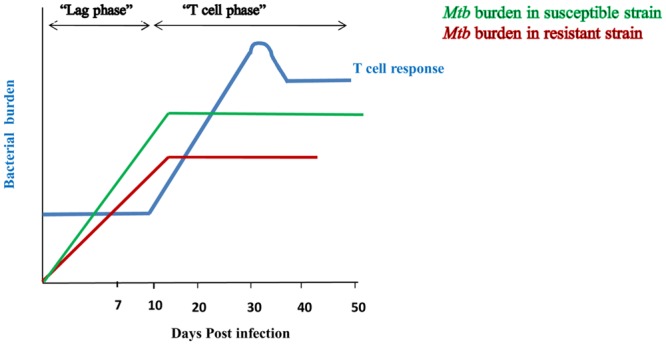
**Innate immunity restricts the bacterial burden during lag phase of T cell response.** Initiation of T cell response occurs after 9–11 days of *Mtb* infection and peaks at 20–25 days (-). Delay in the duration for the generation of effective T cell response is considered as its “lag phase”. Susceptible strain (-) of rabbit shows high bacterial burden during lag phase of T cell response; whereas resistant strain (-) signifies lesser bacterial burden. However, after initiation of T cell response, both the strains restrict *Mtb* growth. It indicates that lesser bacterial burden during lag phase of T cell response in resistant strain of rabbit is due to involvement of innate immunity.

Over the past several decades, research related to defense against *Mtb* was largely focused on the T cells because of their remarkable ability to generate *Mtb* specific immunity, followed by an enduring memory response to counter subsequent infections (Stenger and Modlin, 1999). Undoubtedly, T cells play a crucial role in protection against *Mtb*. However, recent information has chiseled the long belief that T cells are the sentinels in *Mtb* protection, in part due to the substantial lag period between infection and the establishment of specific T-cell responses (Shaler et al., 2012). Recruitment of DCs to the site of infection, followed by their *Mtb* acquisition and transportation toward draining lymph nodes to prime naïve T cells takes 9–11 days after the invasion of the pathogen. Hence, T cell activation occurs after considerable time of infection. This delay between the onset of infection and generation of specific effector T cells provides enough time for *Mtb* to establish an infection (**Figure 1**). Once established, *Mtb* ultimately hampers the antigen processing and priming of naïve T cells (Roberts and Robinson, 2014). Eventually, obstructs the generation and propagation of anti-*Mtb* T cell responses. However, despite the lag phase in T-cell responses, >90% of infected individuals are asymptomatic, raising the possibility of the involvement of other factors in controlling TB.

Lurie’s fundamental studies with resistant and susceptible inbred rabbits proved that the innate response effectively controls the growth of *Mtb* at early times of infection. After 7 days of inhalation of tubercle bacilli, lungs of susceptible animals showed 20–30-fold more bacteria than resistant strain (Lurie, 1964; Arthur et al., 1994) (van Crevel et al., 2002) (**Figure 1**). Protection during the initial phase of infection clearly indicates that T cells are not at the forefront in controlling the infection, but, rather that components of the innate immune system play a pivotal role in generating efficient immunity against *Mtb*. Therefore, it is important to dissect the mechanisms responsible for curbing the growth of *Mtb* during the lag phase of T cell response. Understanding these mechanisms could pave ways in designing novel therapeutic strategies and vaccines to enhance the immune response more efficiently against *Mtb*.

### Why Focus More on Innate Immunity?

In the past, the role of innate immunity was ignored in inducing a protective response against *Mtb*. Recent reports show that the function of innate immunity is even more effective than T cell response against *Mtb* (Fremond et al., 2004; Nicolle et al., 2004). Mice with defective MyD88 signaling show optimal T cell response, yet there is no significant reduction in the lung bacterial burden of *Mtb* challenged mice, compared to wild type (Nicolle et al., 2004). In another study it was shown that MyD88 knockout mice show interferon-(IFN)-γ production in response to mycobacterial antigens but *Mtb* infection became lethal within 4 weeks of post infection with 2 log_10_ higher CFUs in the lung (Fremond et al., 2004). TLR-2 deficient or TLR-9 KO mice show high levels of IFN-γ and tumor necrosis factor (TNF)-α, with high infiltration of CD4 T cells and CD8 T cells in lungs but these mice succumb to *Mtb* infection (Carlos et al., 2009). These reports indicate that innate immunity has a more profound role than to simply assist adaptive immunity. Moreover, an optimal acquired immune response is not sufficient to compensate for defective innate immunity. Collectively, these studies suggest that it is important to dissect out the functions assisted by innate immunity to induce a protective response against *Mtb*.

### Innate Mechanisms Act and Foster T Cells to React against *Mtb*

Based on the recent studies, innate immunity has gained much more impetus due to its profound role in early control of *Mtb* infection and in sustaining the T cell response (Sia et al., 2015). These innate mechanisms represent the target to explore in designing new strategies to control *Mtb*. Taking into consideration all these facts, herein we compile the contribution of the bactericidal mechanisms: autophagy, apoptosis, inflammasome formation, and nitric oxide (NO) production in limiting the growth of *Mtb* (**Table 1**). Additionally, we will discuss how innate signaling delivered through pattern recognition receptors (PRRs) such as toll like receptors (TLRs), nucleotide binding oligomerization domain like receptors (NLRs) can augment these mechanisms.

**Table 1:** The role of innate immune mechanisms in restricting the survival of *Mtb* during lag phase.

Mechanism	Function	Reference
Autophagy	Provide the alternative route for antigen processing and presentation. Target cytosolic antigen to lysosome for degradation. Overcome the evasion strategy of *Mtb* to inhibit phagolysosome biogenesis.	Jagannath et al., 2009; Cooney et al., 2010
Apoptosis	Facilitates the presentation of antigen to CD8 T cells. Restrict the bacterial burden.	Winau et al., 2006; Andersson et al., 2014
Inflammasome	Involved in maturation of IL-1β and IL-18.	Fremond et al., 2007
Nitric oxide (NO)	Intracellular killing of pathogen. Regulate IL-1β secretion to control inflammation.	Flesch and Kaufmann, 1991; Chan et al., 1992; Nicholson et al., 1996; Akaki et al., 1997; MacMicking et al., 1997; Rich et al., 1997; Kuo et al., 2000; Nathan and Shiloh, 2000; Chan et al., 2001; Ciccone et al., 2003; Sharma et al., 2004; Lamichhane, 2011

### Autophagy

Autophagy is evolved as a stress response that endows cells with a capability to adjust their biomass and turn over constituents at the time of starvation. It targets the cytoplasmic material, including macromolecules, organelles, and cells undergoing unscheduled apoptosis to lysosomes for degradation, thus periodically cleaning their interiors. Furthermore, autophagy has crucial roles in various biological processes, which include aging, development, degenerative diseases, and cancer (Huang and Brumell, 2014; Jiang and Mizushima, 2014). In addition, it also helps in elimination of pathogens, which exploit the cytosolic compartment for their regular life cycle, or those that are evolved with the capability to arrest phago-lysosome biogenesis (Flannagan et al., 2009). Autophagy is initiated with the sequestering of pathogens intracellularly to form a double membrane envelope that is known as autophagosome. Autophagosome fuses with lysosomes to form autolysosome to degrade pathogens. Thereby, autophagy facilitates the trafficking of mycobacteria to the lysosome for degradation (Gutierrez et al., 2004; Deretic et al., 2009). Similar results are reported in the case of BCG. In addition, autophagy also transports a large proportion of ubiquitinated proteins to lysosomes and augments the bactericidal capacity of lysosomal fraction (Alonso et al., 2007). *Mtb* escapes the immune mechanism by neutralizing the acidification of phagosomes (Deretic et al., 2006; Russell, 2011). Autophagy overcomes this *Mtb* evasion strategy by targeting phagosomes containing bacterium to lysosomes (Jo, 2013). Thus, autophagy provides an additional barrier to neutralize an attempt made by the mycobacterium to manipulate phagosomes maturation.

Noteworthy, autophagy processes bacteria for degradation within early hours of infection, as evidenced by conversion of autophagy marker LC3-I to LC3-II in *Mtb* infected DCs and Mφs (Khan et al., 2016). This experiment categorically indicates that autophagy guards the host against *Mtb* during the initial phase of infection. Importantly, animals with defective autophagy showed increase in the bacterial burden in lungs of *Mtb* challenged animals despite of predominance of Th17 immunity (Castillo et al., 2012). Furthermore, these animals showed remarkable gross tubercle lesions, in contrast to the smaller infected foci in the lungs of control animals. It signifies that autophagy also aid in preventing excessive inflammatory reactions in the host.

Currently, the only available vaccine for TB is BCG. Nonetheless, BCG has failed to reduce the global TB burden. Interestingly, one of the factors associated with the failure of BCG in TB-endemic areas is BCG ability to hamper the fusion of phagosome with lysosomes (Soualhine et al., 2007; Sun et al., 2007; Gowthaman et al., 2012). This interference in the antigen processing and presentation to T cells, results in defective T cell response. Since autophagy can overcome the problem of phago-lysosome biogenesis, targeting autophagy can substantially contribute to improve efficacy of BCG vaccine (Jagannath et al., 2009). It has been shown in a very elegant study that mice immunized with rapamycin-treated DCs infected with BCG showed enhanced Th1 cells mediated protection when challenged with virulent *Mtb*. Rapamycin induced enhancement in antigen presentation was attenuated when autophagy was suppressed by 3-methyladenine or by small interfering RNA against beclin-1 (Jagannath et al., 2009). Targeting autophagy may open new avenues in boostering the efficacy of BCG or immune response against *Mtb*. Dissecting the host factors that regulates autophagy can help in restricting the growth of *Mtb* and simultaneously improving the processing and presentation of antigen and enhancing T cell immunity.

Autophagy can be induced due to starvation, treatment with IFN-γ or rapamycin (Bento et al., 2015). Additionally, triggering through PRRs has direct correlation with the induction of autophagy. Signaling through TLR-4, TLR-3, TLR-7 and NOD-2 receptor can potently induce autophagy (Delgado et al., 2008; Delgado and Deretic, 2009; Cooney et al., 2010; Yuk et al., 2012). TLR-7 triggering enables Mφs to reduce the survival of *Mtb via* induction of autophagy (Delgado et al., 2008). It was further supported through suppression of autophagy by knocking down beclin and Atg5 through siRNA. Innate triggering through NOD-1 and NOD-2 enhances autophagy induction and reduces the bacterial burden within 6 h of infection (Travassos et al., 2010). Murine immunity related to guanosine tri-phosphate induces autophagy and generate large autolysosomal organelles, as a mechanism for the elimination of intracellular *Mtb*. Furthermore, human Irgm1 ortholog (IRGM) augments autophagy and reduces intracellular bacillary load (Singh et al., 2006). Many evidences have been documented to show the potential for autophagy based therapies to target *Mtb* (Bento et al., 2015). Similarly, exploring innate receptors to augment autophagy could also be one of the strategies to boost the host immunity against *Mtb.* Moreover, it would be beneficial in overcoming the failure associated with BCG vaccine.

### Reactive Nitrogen and Oxygen Intermediates

Nitric oxide and reactive nitrogen intermediates (RNI) are considered potent antimicrobial agents acquired by innate cells. Mφs are the major producer of NO. It is released by inducible nitric oxide synthase (iNOs), a heme-protein that catalyzes the oxidation of L-arginine to NO and citrulline. NO production is a critical defense mechanism in determining the outcome of TB infection, since it reduces the survival of *Mtb* (Rich et al., 1997). This information was corroborated with the result observed in mouse model where abrogation of iNOs activity produces dramatic increase in microbial burden (MacMicking et al., 1997). Further, disruption of iNOs gene expression results in a high rate of *Mtb* dissemination and mortality. Stimulation of innate molecules, such as TLRs or NODs trigger the expression of iNOs, which ultimately kills *Mtb*, as evidenced by colony forming units (CFU) assay (Chan et al., 2001). In the mouse model of TB, NO secretion is well known to be an antimicrobial defense mechanism. However, its role in humans is still controversial. Forthcoming evidences indicate that human Mφs and alveolar epithelial cells upon infection with *Mtb* secrete NO to inhibit the intracellular growth of *Mtb*. Additionally, iNOs and markers associated with NO are highly expressed in the Mφs obtained from broncho-alveolar lavage of TB patients and not healthy individuals (Nicholson et al., 1996). Interestingly, patients infected with multidrug resistant (MDR) strain of *Mtb* produce less NO (Sharma et al., 2004). More startling observation came from the report that chemotherapy appears to cure TB in immune-competent mice but fails to do so in NOS2-deficient animals (Nathan and Shiloh, 2000). It concludes that bactericidal drug uses NO pathway for efficient killing of *Mtb* (Ciccone et al., 2003).

IFN-γ induces the production of NO (Flesch and Kaufmann, 1991). After several days of *Mtb* infection, T cells produce IFN-γ. However, production and action of NO is observed within 3 h and it persists for few days in the circulation (Akaki et al., 1997; Rich et al., 1997). Early production of NO signifies that IFN-γ, which is a potential stimulator for NO release, is not being released by T cells, but instead by the cells of innate immunity such as natural killer (NK) cells and γδ T cells (Sada-Ovalle et al., 2008). It suggests that innate immune cells are responsible for early release of NO to restrict the growth of *Mtb* during the initial period of infection. NO is also reported to regulate the synthesis and release of several pro-inflammatory cytokines including IL-1β, TNF-α, and IL-8, which subsequently affect the production of NO in the feedback loop (Kuo et al., 2000). Microbicidal activity of Mφs is also associated with reactive oxygen intermediates (ROI). However, their role in constraining the growth of *Mtb* is not highly significant (Chan et al., 1992; Akaki et al., 1997; Lamichhane, 2011). Probable reason documented is that although ROI appear immediately upon *Mtb* infection but Mφs cease to produce it within 2 h of infection. Further, ROI also have shorter half-lives.

In essence, the NO kills *Mtb* and as well as augments host immunity against the pathogen is well documented in the literature (Chan et al., 2001). It is important to mention that TLRs signaling contributes substantially in release of NO (West et al., 2011). 1, 25-dihydroxyvitamin D is a potent inducer of NO and suppresses the growth of *Mtb* (Rockett et al., 1998). TLRs triggering enhances the bactericidal activity of Mφs by upregulating the expression of the vitamin D receptor and by inducing the enzyme that catalyzes the conversion of 25-dihydroxyvitamin D3 to active 1, 25-dihydroxyvitamin D leading to the induction of antimicrobial peptide cathelicidin (Liu et al., 2006). Hence, it may be considered as a critical molecule in designing new therapeutic strategies to treat TB. TLR ligands are known to induce NO production in antigen presenting cells (APCs) and NO restricts the growth of *Mtb* (Khan et al., 2015).

### Apoptosis

Apoptosis is commonly known as ‘programmed cell death (PCD)’. It is a phenomenon that occurs when a cell committing suicide confines its cytoplasmic content within membrane bound vesicles named as apoptotic bodies. These membrane bound vesicles express molecules known as ‘eat me or find me’ signals. ‘Eat me’ signals help in the recognition of these unwanted moieties by phagocytic cells (Behar et al., 2011). Furthermore, phagocytic cells remove them through a mechanism known as efferocytosis; the process known to engulf and remove apoptotic cells. Failure in efferocytosis results in the disintegration of apoptotic bodies and release of intracellular contents. This causes inflammation that is known as secondary necrosis (Martin et al., 2012). Importantly, apoptosis makes a crucial contribution to the host immune response and determines the outcome of infection. It abolishes the protected intracellular niche favoring the replication of *Mtb*, thus forcing the bacteria to search for a new habitat. The caspase family of serine proteases are the central molecules responsible for the execution of apoptosis. Apoptosis is classically induced by three pathways. First is through ligation or oligomerization of tumor necrosis factor receptor (TNFR) family. Ligation of cell surface receptor such as TNFR or Fas, results in the subsequent activation of caspases and the induction of apoptotic vesicles. Intrinsic apoptosis occurs in response to oxidative stress, nutrient starvation or intracellular stress, which changes the mitochondrial membrane permeability. It results in the translocation of cytochrome c from mitochondria to the cytoplasm, leading to the activation of caspases. The third pathway is mediated by granzyme B released from cytolytic T cells and NK cells.

*Mycobacterium tuberculosis* induces apoptosis through the classical extrinsic pathway. Encounter of *Mtb* with innate cells such as DCs and Mφs induces the release of TNF-α and triggers apoptosis. Apoptosis limits the replication of *Mtb* by sequestering bacilli in apoptotic vesicles and by activating nearby uninfected Mφs. This phenomenon has been demonstrated through a classical experiment in which uninfected autologous Mφs were cultured with apoptotic or necrotic or non-apoptotic infected Mφs. Interestingly, significant inhibition in the growth of *Mtb* was seen when apoptotic cells were cultured with uninfected Mφs. In coculture experiments, elimination of *Mtb* was anticipated through efferocytosis. Later, antimicrobial effect enacted by naïve Mφs was shown to be contact independent (Hartman and Kornfeld, 2011). Interleukin-1 signaling in naïve Mφs mediates the cross-talk with infected-Mφs. It exhibits NO-dependent antimicrobial activity against bacilli in autolysosomes of heavily infected Mφs (Hartman and Kornfeld, 2011). Noteworthy, the discrepancy occurs in the induction of apoptosis by avirulent *versus* virulent *Mtb* (Chen et al., 2006). Multiple reports indicate that virulent *Mtb* induces necrosis to avoid host defensive strategies, whereas attenuated strain is associated with apoptosis (Chen et al., 2006; Divangahi et al., 2009). Despite comparable amount of TNF-α, cells infected with avirulent strain are more susceptible to apoptosis. It was revealed that difference in level of apoptosis between *Mtb* strains is due to an evasion strategy used by the virulent strain of *Mtb*. Cells infected with virulent *Mtb* secrete more IL-10, which induces the release of TNFR-2. Soluble TNFR-2 forms a complex with TNF-α and downregulates the TNF-α induced apoptosis (Balcewicz-Sablinska et al., 1998). Furthermore, it has been demonstrated by Annexin V binding and intracellular caspase staining that early secretory antigen target (ESAT)-6 of *Mtb* induces apoptosis in human Mφs (Choi et al., 2010). Additionally, the expression profile of apoptotic genes shows up-regulation of anti-apoptotic genes in virulent *Mtb* infected Mφs.

In addition to the restriction of the *Mtb* growth during early phase of infection, apoptosis has a considerable role in the induction of the acquired cellular immune response (Winau et al., 2006). The role of both CD4 T cells and CD8 T cells are well documented in immunity against *Mtb*. However, the mechanism underlying the presentation of antigens to CD8 T cells in context with MHC-I molecules remains enigmatic. Recently, it has been shown that apoptosis of infected Mφs facilitates the release of mycobacterial antigens in apoptotic vesicles, thereby allowing their access to bystander APCs to present antigen to CD8 T cells. Inhibition of apoptotic blebbing using caspase inhibitors, hampers the CD8 T cell response (Winau et al., 2006). Therefore, it may be concluded that triggering of apoptosis can efficiently control the *Mtb* growth at early time points; and at later stages it potentially contributes in the generation of antigen specific CD8 T cells.

Neutrophils are important cells of innate immunity. They play a significant role in imparting protection to *Mtb* (Andersson et al., 2014). These are the first cells to be recruited at the site of infection. Neutrophils phagocytose *Mtb.* Furthermore, *Mtb* infected neutrophils undergo apoptosis and are phagocytosed by Mφs. These Mφs then release TNF-α to form granulomas and control acute *Mtb* infection (Perskvist et al., 2002). Further, inhibition of apoptosis in neutrophils delays the priming of CD4 T cells. Hence it implies that apoptosis plays a decisive role in controlling *Mtb* infection by activating innate as well as adaptive immunity. TLRs induced apoptosis such as TLR-3, 4 has been explored for cancer therapy (Salaun et al., 2007). Interestingly, TLRs show enough potential for triggering apoptosis in *Mtb* infected cells. LpqH, a 19 and 38 kDa lipoprotein of *Mtb* induces the Mφ cell death in TLR-2 dependent manner (Ciaramella et al., 2000; Sanchez et al., 2012). 38 kDa lipoprotein of *Mtb* elicits the TNF-α release in TLR-2 dependent manner and induces apoptosis in infected Mφs (Sanchez et al., 2009). Apoptosis has been shown to improve the efficacy of BCG vaccine. Deletion of the secA2 gene of *Mtb*, which encodes a component of a virulence-associated bacterial protein, triggers the apoptosis of infected cells and enhances the priming of antigen specific CD8 T cells. Vaccination with secA2 deleted *Mtb* mutant induces better protection than BCG against *Mtb* (Boom, 2007; Hinchey et al., 2007). rBCG strain that secretes listeriolysin of *Lysteria monocytogens* induces more efficacious protection than BCG against *Mtb* by facilitating the cross priming by inducing apoptosis (Grode et al., 2005). This evidence indicates that targeting apoptosis could be one of the potential strategies to prevent TB.

Although apoptosis is the well-studied PCD, but it is not the only mechanism responsible for this process. A new form of non-apoptotic PCD has been termed as paraptosis. Insulin like growth factor I receptor has been identified as a molecule involved in inducing paroptosis. It is characterized by cytoplasmic vacuolation, along with mitochondrial swelling, lack of apoptotic morphology, caspase activation and inhibition by caspase inhibitors (Sperandio et al., 2004). A few reports suggest that this form of cell death is driven by an alternative caspase-9 activity that is Apaf-1-independent (Sperandio et al., 2000). Since, paraptosis follows the pathway different from apoptosis, it could be a novel therapeutic target to kill pathogens that inhibits apoptosis. Little is known about the effect of paraptosis on the immune system and moieties involved in it. A few apoptotic inducers have been shown to elicit paraptosis (Amarante-Mendes et al., 1998). Nothing is known about its role in TB. In the future, it may be an interesting line of investigation to understand the contribution of paraptosis in limiting the *Mtb* growth.

### Inflammasome

*Mycobacterium tuberculosis* activates the cascade of events mediating the release of an array of pro-inflammatory cytokines such as IL-6, IL-12, and TNF-α that play a defensive role in eliciting innate immunity (Cooper et al., 2011). Similarly, IL-1β and IL-18 have an influential role in imparting protection to *Mtb*. IL-18 enhances the production of IFN-γ and its abrogation results in less IFN-γ release and impaired NK cell function (Kawakami et al., 2000). Simultaneously, IL-1R1-deficient mice show 2-log increase in bacterial load in the lung and necrotic pneumonia within 4 weeks of *Mtb* exposure. It is notable to mention that cell mediated immunity (CMI), which is considered the hallmark of protection against *Mtb* is not sufficient in restricting bacterial burden in IL-1R deficient mice, despite efficient pulmonary CD4 T cell and CD8 T cell responses (Fremond et al., 2007).

Unlike other proinflammatory cytokines, IL-1β and IL-18 are synthesized as precursors known as pro-IL-1β and pro-IL-18 (Sansonetti et al., 2000). Multiple signaling pathways triggered through TLRs and cytokines result in the transcription of pro-IL-1β and pro-IL-18. However, their maturation requires processing by active caspases. Distinct caspases regulate the apoptosis and maturation of IL-1β and IL-18. Caspase-1 regulates the maturation of IL-1β and IL-18 (Dinarello, 2006). Importantly, release of IL-1β and IL-18 is highly regulated phenomenon, which is dependent on the activation of caspase-1 and its homolog by multimeric protein complex termed as inflammasomes (Vladimer et al., 2013). These complexes are critical in the proteolytic processing of pro-IL-1β and pro-IL-18 into their active form (Netea et al., 2010; Briken et al., 2013). The inflammasome is classically composed of NOD like receptors (NLRs), the adaptor molecule PYCARD/ASC, and pro-caspase-1, which when proteolyzed to caspase-1 provides the enzymatic activity of the inflammasome. Pro-caspase-1 forms the core of the inflammasome. However, the constitution of NLRs within the inflammasome varies according to the type of pathogen involved. The NLR family members NALP3, NAIP5, or IPAF and the adaptor apoptosis speck-like protein (ASC) are involved in caspase-1 activation. Inflammasome plays an important role in host defense against *Mtb*, since mice deficient in IL-1 receptor (IL-1RI), IL-1β or IL-18 are more susceptible to infection with *Mtb*. Furthermore, a defect in ASC adaptor protein shows the exacerbation of disease without restricting the *Mtb* growth. Early secreted antigenic target protein 6 kilodalton secretion system (ESX)-1 encoded in RD-1 region of *Mtb* promotes the release of IL-1β by inflammasome activation. ESX-1 mediated inflammasome formation depends on host NLRP-3 and ASC protein (Mishra et al., 2010). RD-1 deficient *Mtb* fails to induce a strong activation of caspase-1 resulting in inefficient secretion of IL-1β and IL-18. This observation signifies that the failure of BCG to mount optimal protection against *Mtb* is due to absence of RD-1 dependent induction of IL-1β and IL-18 (Kurenuma et al., 2009). Interestingly, treatment with exogenous IL-18 reduces the bacterial load in mice. Recently, viral and bacterial RNA have been shown to trigger NLRP3 and activate inflammasome (Mitoma et al., 2013). It suggests that prophylactic strategies employing recombinant BCG expressing innate ligands, which are efficient in inducing inflammasome formation, can boost its protective efficacy against TB. *Mtb* genes Rv0198c (zmp1), plays a critical role in preventing caspase-1-dependent activation and secretion of IL-1β. zmp1-deleted *Mtb* triggered activation of the inflammasome, resulting in increased release of IL-1β, enhanced maturation of *Mtb* containing phagosomes, improved mycobacterial clearance by macrophages, and reduction in bacterial load in the lungs of *Mtb* aerosol-infected mice (Master et al., 2008). Zmp1 is an important virulence determinant and represents a potentially useful drug target. Furthermore, it has been shown that binding of vitamin D induces IL-1β secretion and prevent infection. This information supports the idea of exploiting vitamin D in clinical trials against *Mtb* (Verway et al., 2013).

Inflammasomes are also reported to play an important part in amplifying the adaptive immune response. Importantly, inflammasome processed IL-1β promotes the differentiation of naïve CD4 T cells to Th17 subtype. It synergizes with IL-6 and promotes Th17 cell development *via* up-regulation of key cytokine IL-17, transcription factors, IRF4 and RORγt. Furthermore, IL-1β can coordinate with IL-6 and IL-23 in the absence of TGF-β signaling to induce pathogenic Th17 cells (Ghoreschi et al., 2010). In addition to Th1 cells, Th17 cells also play a cardinal role in generating anti-*Mtb* response. IL-17 induces the expression of chemokines that results in the recruitment of various cells to the site of infection. Furthermore, memory Th17 cells promote rapid migration of Th1 cells by enhancing the expression of chemokines (Khader and Cooper, 2008). Toll-like receptors and NOD-2 expressed on APCs are responsible for the induction and release of cytokines like IL-6, TGF-β, and IL-12 that are responsible for the differentiation of Th17 cells and Th1 cells, respectively (Khan et al., 2016). Hence immunotherapies involving agonists of innate immunity can be explored in the generation of protective immunity against *Mtb* (Chodisetti et al., 2015). The above showcased points indicate that innate immunity efficiently controls the *Mtb* growth during early phase of infection. Moreover, it creates a platform for adaptive immunity.

## Conclusion

Continuous efforts are undertaken to generate an effective vaccine against TB. However, a possible candidate that can achieve the WHO-STOP-TB program has not yet been formulated. Eleven candidate vaccines are currently in clinical trials. Failure of BCG to protect against *Mtb* warrants a serious attempt to reinvigorate BCG potency for inducing optimal immune response (Singh et al., 2010; Gowthaman et al., 2011, 2012).

Recently, innate immunity has emerged as a cornerstone in limiting the growth of *Mtb* (Fremond et al., 2004; Nicolle et al., 2004; Carlos et al., 2009). Innate immunity not only initiates series of events to assist adaptive immunity but also restricts the growth of TB bacilli at the initial phase of infection. Nonetheless, failure of innate killing mechanisms results in unobstructed growth of *Mtb* and provides enough opportunity for the pathogen to breach the barrier of the immune system. It indicates that targeting innate immunity is a judicious approach to consider, while designing vaccines or therapeutics. Inefficiency of innate immunity provides an opportunity for unimpeded *Mtb* growth. Later, the *Mtb* conquers the adaptive immunity. Adequate innate immunity is capable of restricting the growth of *Mtb* during the “lag phase” of T cell response. Limiting the growth of *Mtb* during the initial phase of infection provides enough time for T cells to reach the site of infection and curtail *Mtb* replication. However, impaired innate immunity is incompetent in curbing the proliferation of *Mtb*. It results in unhindered growth of *Mtb*, which ultimately interferes in the activation of adaptive immunity. Biological therapies involving innate ligands for TLRs and NLRs will benefit the quest for novel treatment modalities for TB. We speculate that mycobacterial vaccines engineered with ligands for PRRs may enhance the potency of innate immunity to limit the *Mtb* growth and sustain the adaptive arm of immunity.

## Author Contributions

Conception and design of the work: JA, NK. Drafting of manuscript: JA, NK, AV, SJ.

## Conflict of Interest Statement

The authors declare that the research was conducted in the absence of any commercial or financial relationships that could be construed as a potential conflict of interest.

## Abbreviations

BCG: *Bacillus Calmette–Guerin*
DCs: dendritic cells
*Mtb*: *Mycobacterium tuberculosis*
PCD: programmed cell death
TB: tuberculosis
